# Supplementary material for: Long-Term Exposure to Decabromodiphenyl Ether Promotes the Proliferation and Tumourigenesis of Papillary Thyroid Carcinoma by Inhibiting TRß
Source: Cancers (Basel). 2022 Jun 2;14(11):2772. doi: 10.3390/cancers14112772 (PMC9179891; doi:10.3390/cancers14112772)
Supplement: Supplementary file 1 [file cancers-14-02772-s001.zip › cancers-1708163-supplementary.pdf]

cancers

MDPI

Supplementary Materials

Long-Term Exposure to Decabromodiphenyl Ether Promotes the Proliferation and Tumourigenesis of Papillary Thyroid Carcinoma by Inhibiting TRß

Xinpei Wang [1,2], Xiujie Cui [1,3], Qian Zhao [2], Feifei Sun [1], Ru Zhao [1], Tingting Feng [1], Shaofeng Sui [4], Bo Han [1,5] and Zhiyan Liu [2,*]

[1] The Key Laboratory of Experimental Teratology, Ministry of Education and Department of Pathology, School of Basic Medical Sciences, Shandong University, Jinan, Shandong 250012, China; wang-xinpei@mail.sdu.edu.cn (X.W.); cuixiujie0826@163.com (X.C.); 201820521@mail.sdu.edu.cn (F.S.); 201715012@mail.sdu.edu.cn (R.Z.); 202190000038@sdu.edu.cn (T.F.); boh@sdu.edu.cn (B.H.)
[2] Department of Pathology, Shanghai Jiao Tong University School of Medicine Affiliated Sixth People's Hospital, 600# Yishan Rd, Shanghai 200233, China; zhaoqian9168@163.com
[3] Department of Pathology, The Second Hospital, Shandong University, Jinan, Shandong 250033, China
[4] Department of Environmental Health, Division of Health Risk Factors Monitoring and Control, Shanghai Municipal Center for Disease Control and Prevention, State Environmental Protection Key Laboratory of Environmental Health Impact Assessment of Emerging Contaminants, 1380 West Zhongshan Road, Shanghai 200336, China; suishaofeng@scdc.sh.cn
[5] Department of Pathology, Qilu Hospital, Shandong University, Jinan, Shandong 250012, China
* Correspondence: zhiyanliu@shsmu.edu.cn; Tel.: +86-189-3017-2295

pTRE-TA promoter sequences: (vector: pGL3)
GAGCTCTGACCCCAGCTGAGGTCAAGGTCACAGGAGGTCAAGGTCATGACCTT-GACCCCAGCTGAGGTCAAGGTCACAGGAGGTCAAGGTCATGACCTTGACCCCA GCTGAGGTCAAGGTCACAGGAGGTCAAGGTCATGACCTTAGAGGGTATA-TAATGGAAGCTCCTCGAG

The sequences of the primers:
THRB, 5′-GAA CAG TCG TCG CCA CAT CTC-3′ (F), 5′-TCT TGC TGT CAT CCA GCA CCA AATC-3′ (R); GAPDH, 5′-ACC CAT CAC CAT CTT CCA GGA G-3′ (F), 5′-GAA GGG GCG GAG ATG ATG AC-3′ (R).

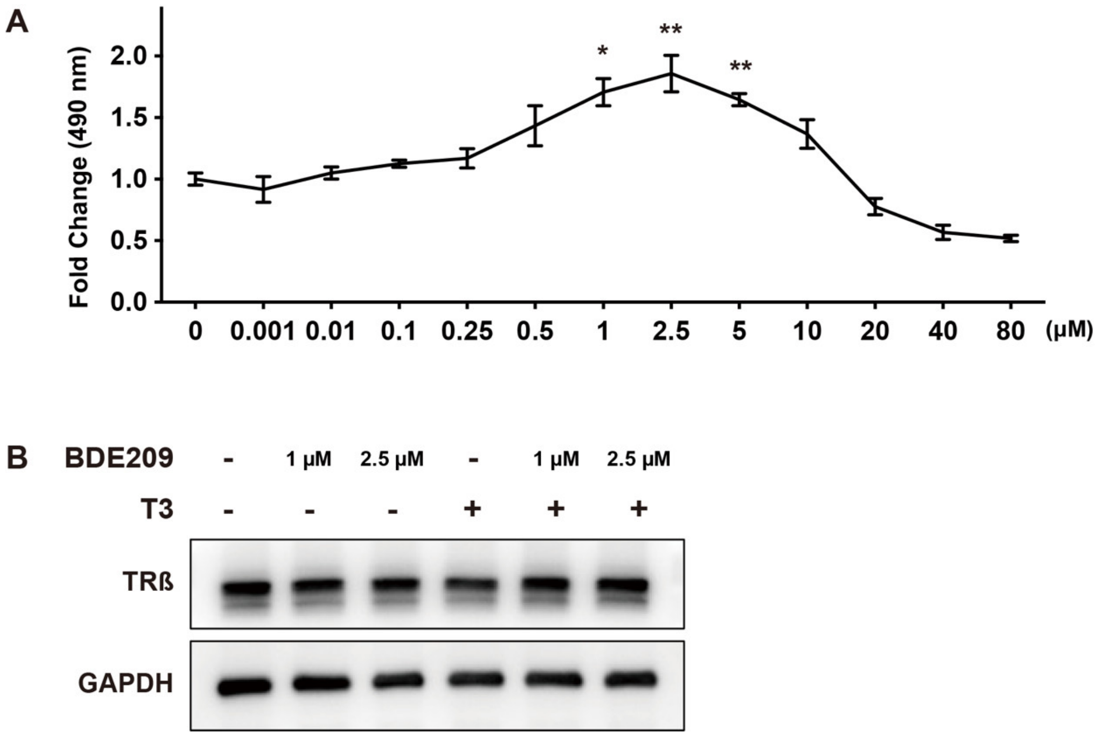

Figure S1. Short-term environmentally relevant doses BDE209 promoted Nthy-ori 3-1-cell proliferation but the TRß expression did not change. **(A)** The cell proliferation of Nthy-ori 3-1 following exposure to different concentrations of BDE209 for 48 h. **(B)** The expression of TRß protein after treatment with different concentration of BDE209 and T3 for 48 h. Data represents the mean ± SD of three separate determinations in triplicate. * $p < 0.05$, ** $p < 0.01$, compared with the control group.

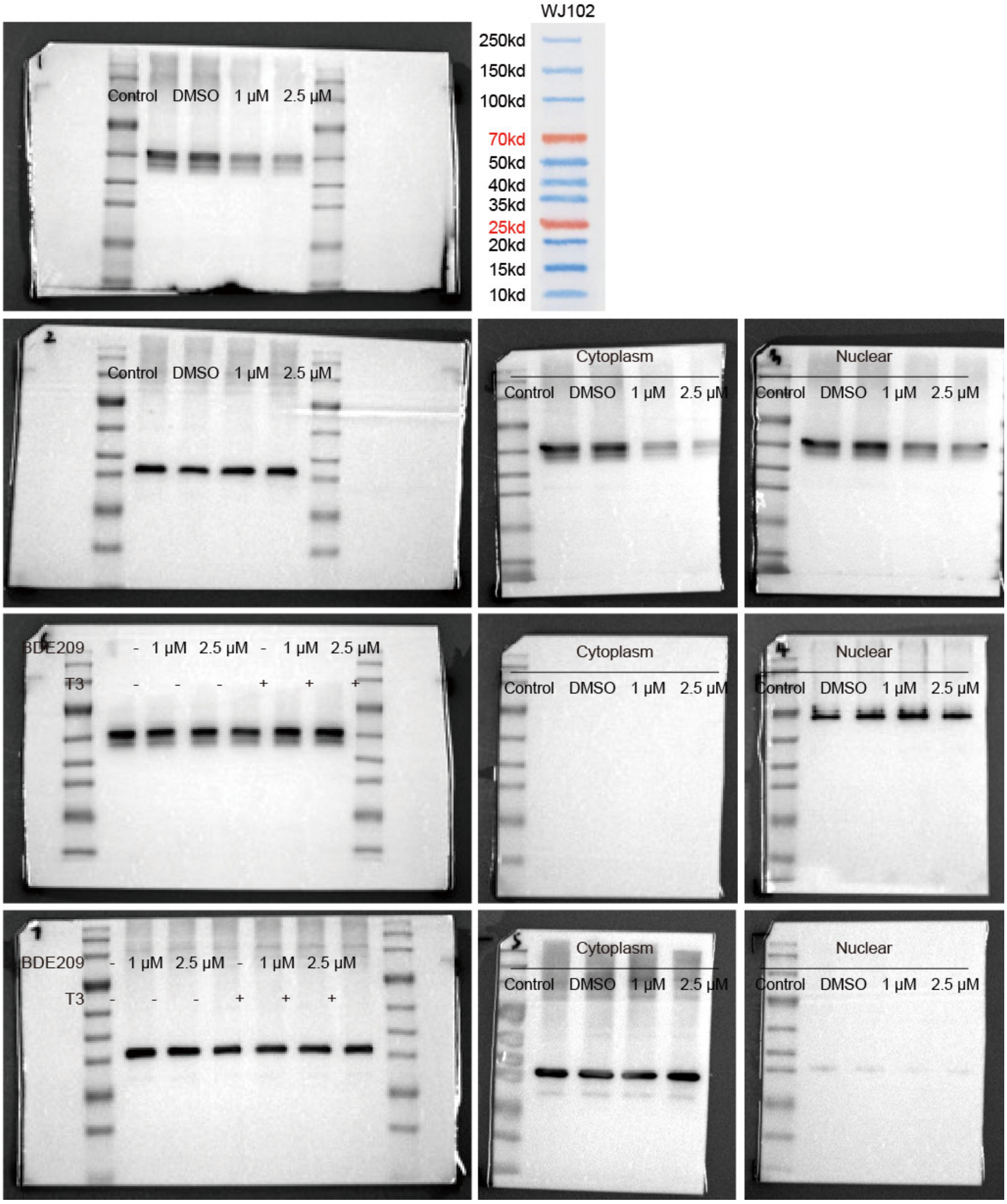

Figure S2. Full images of Western blot.
